# Millimeter-Wave Bat for Mapping and Quantifying Micromotions in Full Field of View

**DOI:** 10.34133/2021/9787484

**Published:** 2021-08-16

**Authors:** Yuyong Xiong, Songxu Li, Changzhan Gu, Guang Meng, Zhike Peng

**Affiliations:** ^1^State Key Laboratory of Mechanical System and Vibration, School of Mechanical Engineering, Shanghai Jiao Tong University, Shanghai 200240, China; ^2^MoE Key Lab of Design and Electromagnetic Compatibility of High Speed Electronic System, and MoE Key Lab of Artificial Intelligence, AI Institute, Shanghai Jiao Tong University, Shanghai 200240, China

## Abstract

Echolocating bats possess remarkable capability of multitarget spatial localization and micromotion sensing in a full field of view (FFOV) even in cluttered environments. Artificial technologies with such capability are highly desirable for various fields. However, current techniques such as visual sensing and laser scanning suffer from numerous fundamental problems. Here, we develop a bioinspired concept of millimeter-wave (mmWave) full-field micromotion sensing, creating a unique mmWave Bat (“mmWBat”), which can map and quantify tiny motions spanning macroscopic to *μ*m length scales of full-field targets simultaneously and accurately. In mmWBat, we show that the micromotions can be measured via the interferometric phase evolution tracking from range-angle joint dimension, integrating with full-field localization and tricky clutter elimination. With our approach, we demonstrate the capacity to solve challenges in three disparate applications: multiperson vital sign monitoring, full-field mechanical vibration measurement, and multiple sound source localization and reconstruction (radiofrequency microphone). Our work could potentially revolutionize full-field micromotion monitoring in a wide spectrum of applications, while may inspiring novel biomimetic wireless sensing systems.

## 1. Introduction

Bats are arguably the most unusual mammal with a remarkable capability of ultrasonic echolocation, enabling them to perceive the environment and preys in complete darkness [1]. With large and complex-shaped ears and sophisticated neural processing, echolocating bats exhibit accurate three-dimensional localization of multiple targets in the full field of view (FFOV) [2–5]. Furthermore, although the neural processing mechanism remains somewhat unclear, bats are particularly notable for perception of small motions (e.g., insects' fluttering wings and frogs' dynamically inflated vocal sac) using short-duration broadband frequency-modulated (FM) calls, which is critical to distinguish and capture preys especially in noisy or cluttered environments [6–9]. It is worth noting that tiny motions are widespread from the natural world to engineering, including heartbeat and bridge vibrations, which carry a wealth of meaningful physical information [10–12]. The accurate and robust perception of tiny motions is significant in a variety of fields, such as healthcare monitoring [13, 14], human-computer interaction [15], Internet of Things [16], and structural health monitoring [17]. Particularly, imaging and quantifying micromotions in a two-/three-dimensional (2D/3D) space is highly desirable for numerous applications from biology to engineering [18–22], and motivating multiple artificial technologies [23–25].

However, current artificial approaches are mainly based on contact sensors (e.g., wearable devices and accelerometers), camera sensing, and laser scanning, each of which suffers from fundamental problems. A network with a mount of accelerometers is generally time consuming and costly and commonly has low accuracy for displacement measurement, while the contact manner has many limitations in practical applications. Camera-based solutions have rich spatial information but suffer low accuracy and high computing load due to sophisticated processing of image stream data. Typically, camera systems also remain challenging with poor lighting conditions and highly dynamic motion visualization. Laser-based approaches, such as the laser Doppler vibrometer, commonly require scanning to achieve planar or spatial micromotion information, which limits measurement synchronization. In addition, they suffer the drawbacks of high cost, large volume, highly specialized setups, and stringent operating environment requirements. Distinct with the common range and velocity detection [26], emerging radar-based radiofrequency (RF) methods can potentially sense and measure micromotions [27–29]. However, they are limited to monitoring only one target or several targets far apart in one-dimensional space, while struggling with tricky clutter interferences [30, 31].

Here, inspired by echolocating bats in terms of micromotion spatial localization and sensing, we have developed a concept of millimeter-wave (mmWave) full-field micromotion sensing (MFMS), a method for noncontact imaging and monitoring tiny motions in FFOV. We refer to this concept as mmWave Bat (mmWBat), which transmits and receives mmWave signals instead of ultrasonic wave signals, allowing artificial system miniaturization and has a large monitoring range. In mmWBat, multiple transmitter and receiver antennas are arranged for measuring azimuth and elevation, mimicking the two powerful ears of bats, which is quite distinct from the existing approaches of radar-based micromotion sensing (the micromotion is commonly measured within the range profile). By emitting and receiving linear-frequency-modulated continuous wave (LFMCW) across multiple sweeps, we establish the fundamental principle of interferometric phase evolution tracking from range-angle joint dimension, enabling the mmWBat to map and quantify micromotions in FFOV simultaneously and accurately. To illustrate the versatility and appealing advantages of the mmWBat, we demonstrate three disparate applications from biology to engineering: multiperson vital sign monitoring, full-field mechanical vibration measurement, and multiple sound source localization and recovery (termed RF microphone). Our work provides a revolutionary approach for full-field micromotion monitoring in various fields, while offering new perspectives for mmWave sensing as well as potentially inspiring novel biomimetic wireless sensing systems based on an understanding of the perception mechanisms used by echolocating mammals.

## 2. Concept and Implementation of mmWBat

**Figure 1:**
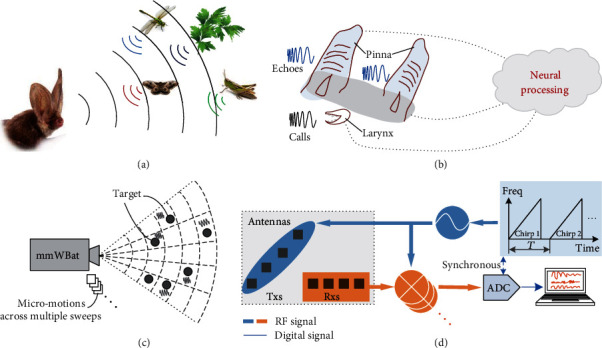
Overall comparison of the echolocating bat and the mmWBat. (a) Schematic of the echolocating bat with multitarget tiny motion perception. (b) Schematic of the biosonar system. (c) Schematic of the mmWBat system with micromotion perception in FFOV. (d) Block diagram of the mmWBat system. Txs: transmitters; Rxs: receivers; ADC: analogue-to-digital converter.

Figure 1 presents a comparison of the echolocating bat (Figures 1(a) and 1(b)) and mmWBat systems (Figures 1(c) and 1(d)) for micromotion sensing in FFOV. The gleaning bat emits a series of ultrasonic calls by its larynx, listens to the echoes by two ears, and processes the contained information of prey and environment via a sophisticated neural system. The bat's sonar system is generally equipped with two large and complex-shaped ears (pinnae and tragi), enabling excellent multitarget spatial localization [4, 5]. Although the echolocating calls can be diverse among different species and tasks, the short-duration, high-frequency, and broadband calls are validated to have great benefits for accurate target localization and classification [7, 32]. The biosonar system of bats is exceptionally sensitive of preys with motions from large to tiny movement, allowing effectively foraging in cluttered environments. Large motions can provide cues with obvious Doppler frequency modulation; however, some species of bats (e.g., the common big-eared bat, *Micronycteris microtis*) have been demonstrated with outstanding capability of micromotion perception in clutter related to informative phase variations, suggesting the neural processing mechanism is just beginning to be understood [6, 7, 9, 33].

Figures 1(c) and 1(d) illustrate the schematic of our biologically inspired mmWBat system, which can distinguish and simultaneously monitor tiny motions of multiple targets in a 2D/3D space. To achieve a long detection range and miniaturize the artificial system, our mmWBat system transmits and receives mmWave signals instead of ultrasonic wave signals. The system consists of a mmWave transceiver with an antenna array, an analogue-to-digital converter (ADC), and a processor, which mimic the biological sonar's emitting and receiving components, neural coding and processing, respectively. A more detailed structure of the mmWBat appears in Supplementary Fig. 1. The antenna array consisting of multiple receiver antennas distributed horizontally and vertically with a certain aperture is configured for mimicking the two large and powerful ears of bats, enabling azimuth and elevation localization. For practical implementation, the multiple-input multiple-output (MIMO) antenna array can be employed to achieve high angular resolution with a small real aperture. Similarly, the mmWBat transmits short-duration, broadband LFMCW mmWave signals with multiple sweeps, which can achieve better range resolution and tracking sensitivity. Note that although bats always shorten their FM calls to avoid overlap of the receiving echoes [34–36], the artificial mmWBat needs to mix the LFMCW broadcasts and the receiving echoes for interference and modulation due to the ultrafast electromagnetic wave propagation speed (i.e., light speed), allowing range detection and sensitive tiny motion perception. Furthermore, the coherence maintenance of the mmWBat system is critical for micromotion perceiving, as the capability of temporal coincidence between sound production and echo reception in the bat's central auditory system, which can be performed by sharing the clock between the ramp generator and ADC or sampling the baseband signal and ramp signal synchronously.

**Figure 2:**
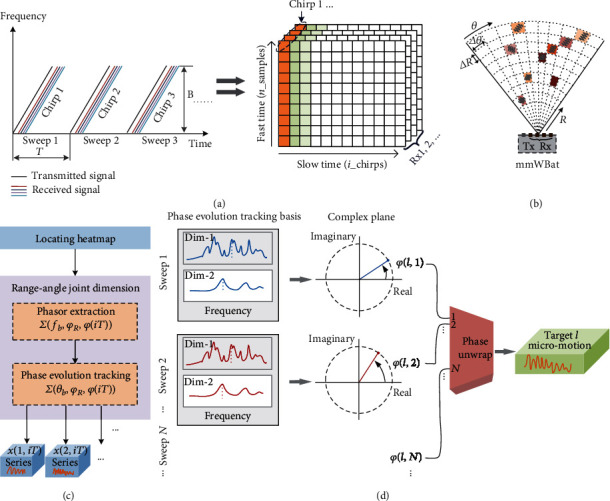
Principle and implementation of the mmWBat. (a) Series of multiple chirps (i.e., sweeps) and data flow. The multichannel baseband signals can be arranged and represented to be a three-dimensional matrix. For per channel, fast time corresponds to the sampling baseband signal of each chirp and slow time corresponds to multiple chirps along time. *T*: sweep period; *B*: transmitted bandwidth. (b) Schematic of multitarget imaging heat map of the mmWBat. Different colors represent different signal strengths. (c) Flow chart of quantifying micromotions of full-field targets. (d) Schematic of micromotion displacement extraction across multiple sweeps in range-angle joint dimension (one target is shown as an example). dim-1: range-dimension; dim-2: angle-dimension.

Here, we demonstrate the proposed fundamental principle of MFMS with the mmWBat system. Figures 2(a) and 2(b) show the schematic of data flow across multiple sweeps with multiple receiver channels and multitarget mapping of the mmWBat. By performing beat frequency estimation of the baseband signal of a certain channel, the mmWBat can commonly achieve the range profile. Although the angular resolution mechanism with digital beamforming depending on an antenna array is well understood, the micromotion quantifying in angle dimension has not been exploited and is considered challenging because of ultrahigh angular resolution requirements, as well as insensitivity to tiny motions. In MFMS, we propose to extract the micromotion information of full-field targets via interferometric phase evolution tracking from the range-angle joint dimension. For ease of illustration, we first consider the single-target scenario. According to the LFMCW ranging principle, the target's reflection produces a beat signal component and the component corresponding to the *i*th chirp can be simply formulated as *S*_*B*_(*iT* + *t*) = *A*(*t*)exp[*j*(2*πf*_*b*_*t* + *φ*_*R*_ + *φ*_*i*_)], where *A*(*t*) is the amplitude of the signal and *f*_*b*_ and *φ*_*R*_ are the beat frequency and fixed phase shift corresponding to the distance between the target and the antenna, respectively. *φ*_*i*_ is the modulated phase which is sensitive to tiny motions. Ignoring the amplitude variation along fast time and performing the beat frequency demodulation, we can represent this component with the phasor *A*exp[*j*(*φ*_*R*_ + *φ*_*i*_)] (see Supplementary Note 1). Generally, we assume the multiple receiver antennas (or equivalent virtual antenna array) of the mmWave sensing system construct a uniformly spaced linear array. According to the geometric relationship of the propagation paths between different antennas (see Supplementary Fig. 2a), the phasor representation of multichannel downconverted baseband signals for the *i*th chirp can be approximated and simplified (see Supplementary Note 1) as

(1) $$\begin{array}{c} A\exp\left(j\varphi_{R}\right)\left[\begin{array}{c} \exp\left(j\varphi_{i}\right)\\ \exp\left(j\left(\frac{2\pi d\sin\theta }{\lambda_{c}}+\varphi_{i}\right)\right)\\ \vdots \\ \exp\left(j\left(\frac{2\pi \left(M-1\right)d\sin\theta }{\lambda_{c}}+\varphi_{i}\right)\right) \end{array}\right], \end{array}$$

where *M* is the total number of equivalent receiver antennas, *d* is the distance between adjacent receiver antennas, *θ* is the incident angle (i.e., the azimuth or elevation angle of the target in FFOV), and *λ*_*c*_ is the wavelength corresponding to the central ramp frequency. It is observed that for the angle-dimensional multichannel phasor signal of each sweep, its frequency is equivalent to *d*sin*θ*/*λ*_*c*_ and its phase is equivalent to *φ*_*R*_ + *φ*_*i*_. Therefore, we show that the key phase evolution (i.e., *φ*_*i*_, *i* = 1, 2, ⋯) information corresponding to the micromotion displacement in range dimension can be reserved and equally transferred to angle dimension. As a result, for mmWave micromotion sensing, we can ultimately estimate and extract the phase history across multiple sweeps from the angle dimension.

Extending to common full-field multitarget scenarios, the angle-dimensional phasors regarding multichannel baseband signals can be expressed as

(2) $$\begin{array}{c} \overset{L}{\underset{l=1}{\sum }}A_{l}\exp\left(j\left(\varphi_{Rl}+\varphi_{il}\right)\right)\left[\begin{array}{c} 1\\ \exp\left(j\left(\frac{2\pi d\sin\theta_{l}}{\lambda_{c}}\right)\right)\\ \vdots \\ \exp\left(j\left(\frac{2\pi \left(M-1\right)d\sin\theta_{l}}{\lambda_{c}}\right)\right) \end{array}\right], \end{array}$$

where *l* = 1, 2, ⋯*L*, *L* is the total number of detectable targets and *θ*_*l*_ is the incident angle of the *l*th target. Therefore, we can simultaneously measure and track the phase evolution corresponding to each target from the converted angle dimension. With the aspect of phasor analysis, it is worth noting that our approach is equivalent to separating and isolating the desired components combined with range dimension and angle dimension, offering great benefits of the challenging clutter interference suppression especially for the mutual coupling issues (see Supplementary Note 2) in real scenarios. In addition, for 3D spatial multitarget localization, the range-azimuth and the range-elevation estimations should be performed in succession. However, when extracting the micromotion displacements of full-field targets, only one of the azimuth and elevation angle dimensions is employed to form the range-angle joint dimension.

For practical implementation, Figure 2(c) shows the flow chart of micromotion measurements of full-field targets with the mmWBat, and Figure 2(d) presents the schematic illustrating how to extract the time-domain displacement signals of a certain target via phase variation estimation in the range-angle joint dimension. In Materials and Methods and Supplementary Fig. 2, we show the basic algorithm of full-field phase evolution tracking. Consequently, according to the interferometric measurement principle, the displacement time series of micromotion of each target can be extracted as *x*(*l*, *iT*) = [*λ*_*c*_(*φ*(*l*, *iT*) − *φ*_*l*_mean_)/4*π*]/cos*ϕ*_*l*_, where *φ*(*l*, *iT*) is the extracted variation phase of target *l* for the *i*th sweep, *φ*_*l*_mean_ is the mean of *φ*(*l*, *iT*), *i* = 1, 2, ⋯, and *ϕ*_*l*_ is the angle between motion direction and the mmWave line-of-sight of target *l*.

## 3. Results

### 3.1. Multi-Person Vital Sign Monitoring with mmWBat

The mmWBat system offers great potential for revolutionizing healthcare solutions in biomedical engineering, which is not wearable, regardless of bad lighting and with complete privacy. Particularly, the example involves noncontact vital sign (i.e., respiration and heartbeat) monitoring is valuable for health disorder evaluation, disease diagnostic, and sleep quality analysis of people, especially for infants and the elderly [37]. Moreover, it is worth noting that the mmWBat can provide a cost-efficient way to simultaneously monitor the vital signs of multiple persons in public places including nursing homes and hospitals. For example, it can perform the health monitoring of a large number of patients in mobile cabin hospitals combating COVID-19, which can save a lot of medical resources, offer long-term comfortable monitoring, and reduce the infection risk of medical workers [38, 39].

The intrinsic principle of vital sign detection with RF sensing is measuring the micromotion of the chest wall caused by cardiopulmonary activity. Although many efforts have been made with microwave radar-based methods, two main challenges remain that prevent the widespread application of this technology. Firstly, since the chest wall movement caused by heartbeat is much smaller than that of respiration, and the movements are averaged with multipoint scattering effect, the current RF-based far-field vital sign monitoring cannot retrieve the heart rate (HR) accurately and reliably in practical scenarios even with careful processing. Secondly, for multiperson (e.g., a couple and their baby in the bed) monitoring scenarios, it requires individuals to be far apart from each other to mitigate mutual interference. The mmWBat system offers an effective approach to address these challenges by full-field micromotion sensing on the multiple body parts of one individual, as well as multiple individuals. For per individual, the mmWBat can automatically locate the chest position of the heartbeat according to the characteristics of heartbeat activity, allowing a great reduction in the average effect of multipoint scattering, which can significantly enhance the desired heartbeat component. For multiple individuals, the mmWBat is attractive to eliminate the tricky mutual interference with sensing in range-angle joint dimension. Note that, since the human body is a flexible continuum, it requires the mmWBat to have relatively high angular resolution for achieving excellent performance.

**Figure 3:**
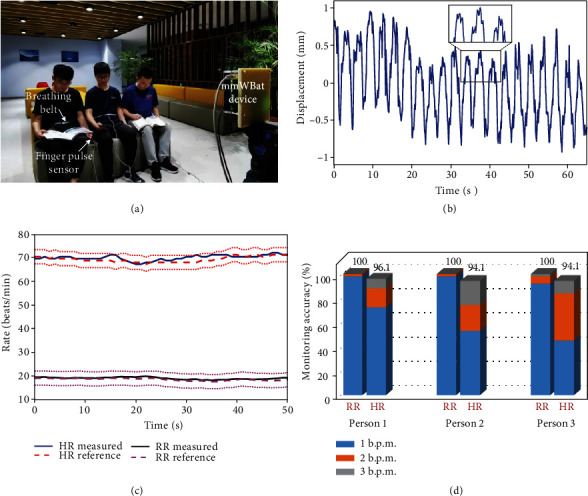
Multi-person vital sign monitoring with the mmWBat. (a) Photograph of experiment scenario. (b), Measured result of the chest wall displacement signal corresponding to the individual on the left. The inset shows the obvious heart beat component. (c) RR and HR tracking results of the individual on the left with comparison of the references. Two dotted lines on both sides of the reference are the defined deviation boundaries of ±3 Beats/Min (i.e., confidence intervals). (d) Vital sign monitoring accuracy of all three individuals. The accuracy is calculated as the percentage of the time when the measured rate falls into the confidence interval, which contains the success rates that the RR and HR are different from the corresponding references of 1 b.p.m., 2 b.p.m., and 3 b.p.m., respectively.

Figure 3 presents the experimental setup (Figure 3(a)) and results (Figures 3(b)–3(d)) of multiperson vital sign monitoring. To mimic a multiuser scenario in a real-world setting, we recruited three volunteers to sit on a couch shoulder by shoulder in a break room and they stay in a quasistatic situation without large body movement. The mmWBat device sensed and monitored their vital signs simultaneously from a distance of about 2 m, and additional details appear in Materials and Methods. As an example, the extracted chest wall displacement signal of the volunteer on the left is shown in Figure 3(b) and that of all individuals are shown in Supplementary Video 1. It is observed that the desired heartbeat component can be well retrieved with full-field micromotion sensing and localization from different body parts. With sliding window processing, Figure 3(c) presents the comparison results of the tracked respiration rate (RR) and HR time series and the corresponding references, which show a good match. The challenging HR tracking, in particular, can be reliably achieved via heartbeat component enhancement sensing with our proposed MFMS method. Moreover, we evaluate the monitoring accuracy of all three individuals in percentage by defining confidence intervals (see Figure 3(d)), with most measurement errors less than ±3 Beats/Min. In addition, the composition of the success percentage with deviation within 1 b.p.m., 2 b.p.m., and 3 b.p.m. is provided to illustrate the agreement performance. These results demonstrate that mmWBat can monitor multiple individuals' vital signs (even with zero separation) and micromotions of different body parts, exhibiting a promising technique for contactless vital sign monitoring.

### 3.2. Full-Field Mechanical Vibration Measurement via mmWBat

**Figure 4:**
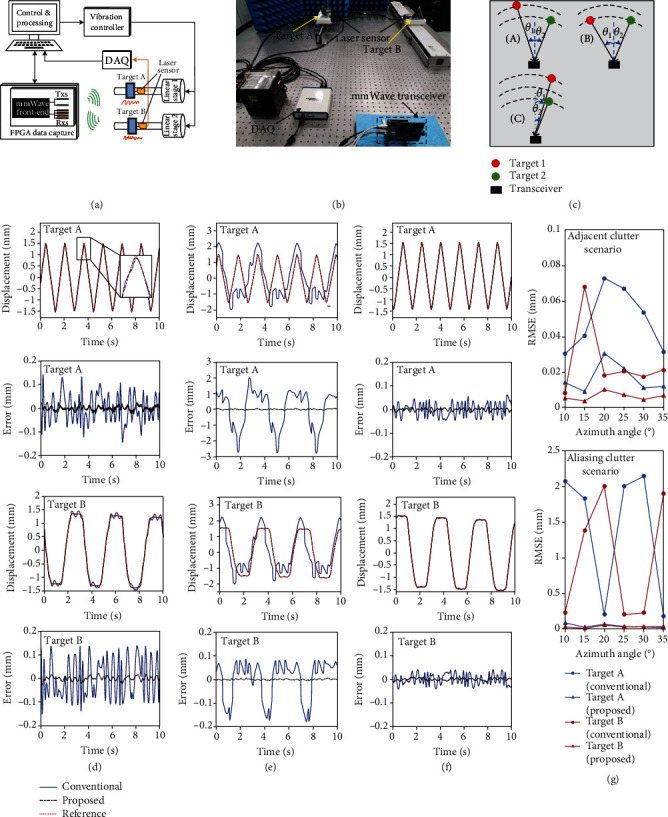
Experimental results of full-field mechanical vibration measurement. (a, b) Experimental setup: block diagram (a) and photograph (b). (c) Schematic illustration of three typical measurement scenarios, i.e., adjacent clutter (A), aliasing clutter (B), and adjacent angle (C). (d–f) Comparison results of displacement tracking of targets A and B regarding three typical scenarios. Adjacent clutter scenario (d) and aliasing clutter scenario (e) with *θ*_1_ and *θ*_2_ about 15° (one angular resolution). Adjacent angle scenario (f) when *θ*_1_ and *θ*_2_ are similar. (g) Comparison of displacement measurement accuracy (i.e., RMSE) of targets A and B with different azimuth angles (i.e., *θ*_1_ ≈ *θ*_2_ ≈ 10°, 15°, ⋯, 35°) corresponding to the first two typical scenarios.

The mmWBat is capable of micromotion mapping and quantification of multiple targets or points simultaneously, thereby creating interesting opportunities for full-field mechanical vibration monitoring. Our approach offers appealing advantages as nonintrusive, full-field, large measurement region and vibration scales (*μ*m to m), along with high reliability in harsh environments, which are highly desirable for structural health monitoring, modal analysis, and machinery diagnostics in mechanical, aerospace, and civil engineering. Here, we validate and compare the performance of the mmWBat and the existing radar-based technique by conducting experiments with various scenarios. Figures 4(a) and 4(b) presents the block diagram and a photograph of the experimental setup. As shown in Figure 4(c), we mimic three typical measurement scenarios (similar to gleaning bats), which can comprehensively examine the significant performance improvement of our approach and highlight the key aspect of clutter interference suppression for mmWave sensing. Additional details concerning the experimental setup appear in Materials and Methods.

Figure 4(d) depicts the comparison result of the typical scenario when the two targets are located in adjacent range bins. In conventional radar-based micromotion sensing, the adjacent clutter interference (caused by the reflection of adjacent objects) is common and has an obvious effect on the measurement accuracy of both targets. It is challenging to evaluate and suppress the adjacent clutter due to the mutual coupling between components (see Supplementary Note 2). However, the mmWBat system can effectively eliminate the adjacent clutter interference from the range-angle joint dimension and obtain accurate displacement measurement results (see Figure 4(d)). In addition, Figure 4(g) illustrates the benefit of the MFMS method when the two targets are placed at different azimuth angles. The root mean square error (RMSE) of measurements is adopted to better evaluate the performance. We show that our approach can achieve much better performance in all cases and the measurement error is stably small, offering an effective strategy to eliminate the tricky clutter interference.

For the scenario when the two targets are located in a same range bin, we also provide corresponding comparison results with a similar form (see Figures 4(e) and 4(g)). Obviously, the existing radar-based technique is difficult or impossible to perform phase evolution tracking due to the severe aliasing problems, which results in failure of multitarget vibration measurement and sensitive to the demodulation frequency. In contrast, the MFMS method can accurately extract the vibration displacement of each target via angle-dimensional demodulation, illustrating the effectiveness and significant advantages of our technique (see the good agreement between reference and measured results). Note that due to the heavy coupling in range dimension, the measurement accuracy in this scenario is indeed slightly lower than the previous scenario, which can be solved by increasing the angular resolution of the mmWBat system. Moreover, we further evaluate the performance when the two targets are close in angle dimension (i.e., targets have similar azimuth angles in our example). Experiment results (see Figure 4(f)) show that although the two targets are adjacent in angle profile, the proposed approach can also achieve better measurement accuracy than the conventional technique. As shown in Figure 4(f), since targets A and B are separated far apart, the current technique can achieve a relatively small measurement error due to the weak interference. However, the proposed method can further improve the measurement accuracy even with adjacent angles, which benefits from the isolation and distribution in range-angle joint dimension. Examples of full-field vibration monitoring with the three typical scenarios illustrated are shown in Supplementary Video 2.

Furthermore, Supplementary Video 3 presents the full-field dynamic monitoring of a scaled bridge (18 cm × 90 cm) under different load conditions, offering an example of vibration and deformation-based structural health monitoring of large structures. With the mmWBat system, we can accurately and remotely measure full-field multipoint displacements of the scaled bridge, creating an effective approach to solve the problem of dynamic deflection monitoring and torsion monitoring. Note that for the torsion monitoring of a long-span bridge in practical applications, owing to the remote monitoring requirement, the system may need to be equipped with more equivalent receiving antennas, which can better eliminate the coupling interference between two parallel measurement points distributed on both sides of the bridge structure.

### 3.3. mmWBat as RF Microphone

We also applied the mmWBat to the field of sound source localization and reconstruction, which is significant and useful in a wide range of contexts such as sound separation and enhancement, target recognition, surveillance, and security [40, 41]. Here, we present an innovative approach to locate and reconstruct multiple sound sources using the mmWBat, creating a unique RF microphone. The key enabler underlying our RF microphone is the proposed MFMS method and the fact that sound is produced by micromotions with magnitude of *μ*m length scale and high frequency. With the mmWBat, we detect and monitor the slight surface movements of sound sources via FFOV sensing. Our approach exhibits several exceptional advantages compared to existing techniques such as microphone array. For example, regardless of the characteristics of multiple sound sources and whether they are coherent, we can achieve high directivity and separation with small size and power, while recovering the high-quality audio signal of each source. Moreover, the RF microphone can easily obtain high-performance range and angular positioning without complex and time-consuming estimation calculations.

**Figure 5:**
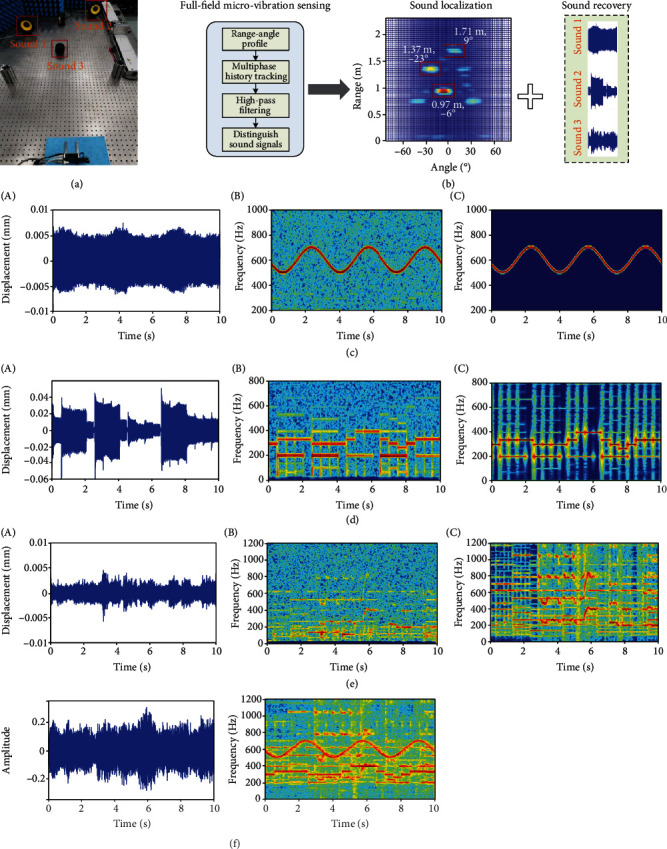
RF microphone. (a) Photograph of the experimental scenario. (b) Schematic illustration of procedures and results of sound source localization and recovery. (c–e) Comparison results of sound recovery regarding sound 1 (c), sound 2 (d), and sound 3 (e). Recovered sound signal is with time-domain (A) and time-frequency (B) representation, and the original sound signal is with time-frequency representation (C). (f) Sound recovery results from a cellphone's microphone.

Figure 5(b) shows the implementation procedure for the RF microphone, which is detailed in Materials and Methods. As a result, we can clearly distinguish the sound sources and obtain their localization information, including their distance and direction, while the extracted micromotion signals can be employed for reconstructing the corresponding sound signals. We validate the feasibility and performance of the RF microphone technique with experiments including three loudspeakers (i.e., sound sources). Figure 5(a) presents the experimental setup, and additional details appear in Materials and Methods. To mimic real scenarios, we put several stationary objects and the three sound source targets in the detection region. As shown in Figure 5(b), we first achieved the range-angle heatmap of all targets and tracked the phase evolution signals corresponding to each possible target based on the MFMS method. Then, we can further process to identify the sound sources (see Materials and Methods and Supplementary Figure 3). Consequently, we were able to extract the range and angle information of all sound source targets accurately (see Figure 5(b), which matches well with the ground truth obtained by rulers), and reconstruct the corresponding audio signal of each source. In the experiment, the three sound sources are different, offering a comprehensive testing scenario. For better evaluation, we show the comparison results of the time-frequency representations of the recovered signal and original audio for each source (see Figure 5(c)–5(e)). It is seen that the RF microphone can effectively separate different sound sources and accurately recover the audio signals. Moreover, as a comparison, the sound signal captured by the traditional microphone of a cellphone is also provided (see Figure 5(f)). Obviously, the conventional microphone cannot separate and isolate different sound sources, which leads to severe sound aliasing. To intuitively evaluate our reconstructed audios with the comparison of the traditional microphone, we recommend listening to Supplementary Video 4.

Note that the RF microphone has a relatively low recovery performance for music sound which generally has many high-frequency components (see Figure 5(e)). This is because the micromotion corresponding to high frequency is ultrasmall, which is susceptible to noise interference. However, the RF microphone can also successfully recover a clear music sound even if high-frequency components are not well preserved (see Supplementary Video 4). Furthermore, for better perception and reconstruction of high-frequency sound, it suggests achieving submicron displacement measurement accuracy which requires high signal-to-noise ratio (SNR). Since the RF microphone has excellent capability for sound source localization, we can further use the beam steering technique to focus on the sound source target of interest, which can well improve the SNR and reduce interference, allowing high-quality broadband sound recovery. Considering that traditional microphones work in response to sound pressure, the RF microphone can be extended to sense and extract the minute motions of other objects stimulated by sound pressure, which is potentially useful for handling scenarios where sound sources are obscured.

## 4. Discussion

Here, we have demonstrated a bioinspired mmWBat system for mapping and quantifying micromotions in FFOV. Its structure is functionally similar to that of the echolocating bat's biosonar system. In particular, we develop a fundamental concept of MFMS, which enables us to image and measure tiny motions of full-field targets simultaneously and accurately, providing significant technological advance compared with the current emerging radar-based approaches. We reveal the intrinsic transfer mechanism of the interferometric phase evolution information between range and angle dimension, allowing full-field micromotion sensing and measuring from the range-angle joint dimension. Meanwhile, the MFMS method also provides an effective strategy for tackling the challenge of tricky clutter interference in mmWave sensing. The potential and appealing advantages of our mmWBat concept are highlighted in three disparate applications, namely, multiperson noncontact vital sign monitoring, full-field mechanical vibration measurement, and multiple sound source localization and reconstruction, which provide novel approaches and insights into addressing challenges of micromotion sensing in various fields. We envision that our approach can revolutionize micromotion monitoring technology with a large monitoring range, full-field synchronous measurement, multiscale visualization, high accuracy, robustness, and low cost. Furthermore, our work opens up new perspectives and motivates interesting research of mmWave sensing, while possibly inspiring novel biomimetic wireless sensing systems with an understanding of the perception mechanisms used by echolocating mammals.

## 5. Materials and Methods

### 5.1. Algorithm of Full-Field Phase Evolution Tracking

The basic algorithm can be implemented with the following two main steps:

1. Full-field target localization and phasor index

As shown in Supplementary Fig. 2b, we first choose a certain (e.g., the first) sweep multichannel baseband signal **H** = [**S**_1_, ⋯**S**_**m**_, ⋯**S**_**M**_], where **S**_**m**_(*m* = 1, ⋯*M*) denotes the corresponding *m*th channel signal and then perform the calculation with a two-dimensional fast Fourier transform (FFT). Specifically, we first calculate the FFT for each channel baseband signal (i.e., each column of **H**) to obtain the range-dimensional phasor matrix **H**_**f**_. Then, we calculate the FFT for each row of **H**_**f**_ (i.e., phasors of multiple channels) and denote the obtained matrix as **H**_**ff**_. Note that to refine the spectrum and reduce the fence effect, we can apply the commonly used zero-padded FFT for practical calculations.

Next, we perform a simple peak detection with ∑_*p*=0_^*P*−1^*abs*(*S*(*k*, *p*)), where *S*(*k*, *p*) is the element in row *k* and column *p* of matrix **H**_**ff**_, *abs*(·) denotes taking the complex magnitude, and *P* is the total column number of **H**_**ff**_. Accordingly, we can achieve the located range bins and the peak index *k*_*l*_(*l* = 1, ⋯) of full-field targets. Similarly, the located angle bins and the corresponding index *p*_*l*_(*l* = 1, ⋯) can be obtained by directly employing the magnitude peak detection on row *k*_*l*_ of matrix **H**_**ff**_. The obtained phasor corresponding to the desired component of each target by FFT and index search has a certain error with the ground truth, but fortunately, it has no effect on the phase variation tracking (see Supplementary Note 2 for details).

(2) Full-field phase evolution estimation

When the heatmap and the corresponding phasor indexes of full-field targets are obtained, the phase evolution of each target can be estimated as

(3) $$\begin{array}{c} \varphi \left(l,iT\right)=\arg\left[\overset{M-1}{\underset{m=0}{\sum }}\overset{N-1}{\underset{n=0}{\sum }}s_{i}\left(n,m\right)e^{-j\left(2\pi {nk}_{l}/N_{z}\right)}e^{-j\left(2\pi {mp}_{l}/M_{z}\right)}\right], \end{array}$$

where *φ*(*l*, *iT*) denotes the estimated initial phase corresponding to the *i*th sweep period of the *l*th target, arg[·] denotes taking the phasor angle, *s*_*i*_(·) is the multichannel baseband signal of the *i*th sweep period with *N* rows and *M* columns, and *N*_*Z*_ and *M*_*Z*_ are the FFT sizes of the first and second FFT calculations in the previous step, respectively. In addition, due to the calculated phase angle that always lies between ±*π*, it is essential to perform the unwrap procedure for practical use.

### 5.2. Experimental Setup and Implementation

#### 5.2.1. Construction of the mmWBat System Prototype

The mmWBat system prototype built consists of a commercial mmWave transceiver, a data capture card, and a laptop. To achieve different angle resolution capacity, we adopted two types of mmWave transceivers: (i) AWR1443, Texas Instruments, which mainly includes microcontroller, single-chip front-end (Tx power: 12 dBm, carrier frequency: 77-79 GHz are used) and onboard antennas (2 Txs and 4 Rxs are used) and (ii) AWR1243P cascade, Texas Instruments, which mainly consists of 4-chip AWR 1243P. The data capture card streams the transceiver raw data over Ethernet to the laptop. The mmWBat system works in LFMCW mode with sawtooth modulation. We use software (mmWave Studio) for transceiver parameter setting, data acquisition, and control. The raw data is saved and processed offline using MATLAB R2017b.

#### 5.2.2. Setup of Multi-person Vital Sign Monitoring Experiments

Three volunteers are asked to sit on a couch shoulder by shoulder and breathe normally. During experiments, they are in a state of daily behavior (i.e., reading books or using their cell phones) in quasistatic situations. A breathing belt and a finger pulse sensor are adopted for each individual, which provide the references of RR and HR time series, respectively. The reference signals are captured by a DAQ device (USB-6210, National Instruments), which is synchronous with the mmWBat system. The second type of mmWBat system prototype is employed in this application, and the main parameters of the mmWBat system are that the transmitted bandwidth is 3 GHz and the sweep cycle is 10 ms.

#### 5.2.3. Setup of Full-Field Mechanical Vibration Measurement Experiments

In these experiments, two corner reflectors are employed as the targets. They are mounted on two linear stages and controlled to achieve different vibration movements (target A: triangular pattern, target B: sinusoidal pattern), respectively. The mimicked vibration device includes two linear stages and a controller with a LabVIEW control interface. To mimic the three typical measurement scenarios, we place target A and the mmWave transceiver on an optical table in different positions. During the experiments, we utilize two laser displacement sensors (LK-G80, Keyence) to provide the ground truths of the two target displacements, respectively. The vibration signals measured by laser sensors are captured by a DAQ device (USB 4431, National Instruments), which is synchronous with mmWBat raw data acquisition. The first type of mmWBat system prototype is employed. With the built system, we employ 2Tx-4Rx antenna configuration with time division multiplexing. The sweep cycle is set to 4 ms, and the transmitting bandwidth is set to 2 GHz (i.e., range resolution is 7.5 cm).

#### 5.2.4. Implementation Procedure for the RF Microphone

First, we achieve the range-angle profile of the monitoring region illuminated by the mmWave beam. Then, we identify the sound source targets depending on the micromotion characteristics of the sound signals. To quickly locate and reduce the amount of calculation, we extract the phase evolution signal of each possible target along slow time according to the range-angle heatmap. To eliminate the interference of low-frequency movement objects and possible undesirable minor phase shift across sweeps due to the temperature drift of hardware, we apply the high-pass filtering (finite impulse response high-pass filter with a cut-off frequency of 40 Hz) to the extracted phase evolution signals. The key consideration for sound localization via mmWBat is to distinguish the sound source targets from other likely static objects. Since the phase history corresponding to a static object is basically broadband thermal noise, there is no obvious frequency component. In contrast, the sound source target usually has rich frequency components which change dynamically. Therefore, we propose to evaluate the time-varying sparseness (TVS) of the amplitude spectrum of each phase history signal with a sliding window. Specifically, we perform an FFT on each window and calculate the ratio of the peak value to the average value of the obtained amplitude spectrum. Then, we distinguish the sound source targets and others with the indicator TVS = *ar*(*t*)_mean_ + *br*(*t*)_std_, where *a* and *b* are weight coefficients (which are set to 1 and 2, respectively, in our implementation due to the obvious time-varying of sound signal spectrums), *r*(*t*)_mean_ and *r*(*t*)_std_ are the mean and standard deviation of *r*(*t*) (i.e., the calculated ratio time series), respectively. If the indicator is larger than the threshold (empirically set to 10), we consider it a sound signal component.

#### 5.2.5. Setup of RF Microphone Experiments

The mmWBat device employed here is the same as in the full-field mechanical vibration measurement experiments. The difference in parameter settings is that the sweep cycle is set to 0.2 ms (i.e., micromotion displacement sampling frequency is 5 kHz). In the experiments, all targets are placed on an optical table (length: 2 m, width: 1.5 m). In terms of the three sound source targets, source 1 is driven by an arbitrary waveform generator which outputs voltage signals with sinusoidal modulation, source 2 is driven and input by a computer and a power amplifier with a tone signal, and source 3 is input to music signals via Bluetooth. The traditional microphone of a cellphone (P30, Huawei) captured the audio signal via a sound recorder app, and the cellphone is placed next to the mmWave transceiver.

#### 5.2.6. Signal Processing Implementation of Experimental Validations

According to the basic algorithm of full-field phase evolution tracking, when performing 2D FFT for multichannel baseband signals, we used the zero-padded FFT technique with *N*_*z*_ = 2*N* and *M*_*z*_ = 180. For multiperson vital sign monitoring experiments, after extracting the chest wall displacement time series, we performed the RR and HR tracking by using the sliding window technique (window length: 15 s, step size: 1 s). For each window, the RR is estimated by autocorrelation analysis after band-pass filtering (0.1-0.9 Hz) and the HR is estimated by first-order differential enhancement analysis. For RF microphone experiments, the time-frequency representations of all recovered and original sound signals are achieved by short-time Fourier transform (STFT) with a sliding window size of 1024.

## Data Availability

The supplementary materials contain additional data needed to evaluate the conclusions of the paper. All other data used to support the findings of this study are available from the corresponding author upon request.

## Supplementary Materials

Supplementary Materials Note S1: phasor representation of multichannel baseband signals. Note S2: principle of clutter interference elimination. Figure S1: block diagram of the architecture of the mmWBat system. Figure S2: schematic of the basic algorithm of full-field phase evolution tracking. Figure S3: comparison results of the calculated ratio curves of the estimated phase evolution signals corresponding to three sound sources and three static objects in the RF microphone experiment. Video S1: multiperson noncontact vital sign monitoring. Video S2: full-field vibration measurement of two targets with three typical scenarios. Video S3: example of full-field dynamic monitoring of a scaled bridge. Video S4: multisource sound recovery of RF microphone.
