# Supplementary material for: Millimeter-Wave Bat for Mapping and Quantifying Micromotions in Full Field of View
Source: Research (Wash D C). 2021 Aug 16;2021:9787484. doi: 10.34133/2021/9787484 (PMC8385533; doi:10.34133/2021/9787484)
Supplement: Supplementary Materials — Note S1: phasor representation of multichannel baseband signals. Note S2: principle of clutter interference elimination. Figure S1: block diagram of the architecture of the mmWBat system. Figure S2: schematic of the basic algorithm of full-field phase evolution tracking. Figure S3: comparison results of the calculated ratio curves of the estimated phase evolution signals corresponding to three sound sources and three static objects in the RF microphone experiment. Video S1: multiperson noncontact vital sign monitoring. Video S2: full-field vibration measurement of two targets with three typical scenarios. Video S3: example of full-field dynamic monitoring of a scaled bridge. Video S4: multisource sound recovery of RF microphone. [file 9787484.f1.zip › Supplementary materials.pdf]

Supplementary Materials

Millimeter-wave bat for mapping and quantifying micro-motions in full field of view

Yuyong Xiong[1], Songxu Li[1], Changzhan Gu[2], Guang Meng[1], Zhike Peng[1*]

[1]State Key Laboratory of Mechanical System and Vibration, School of Mechanical Engineering, Shanghai Jiao Tong University, Shanghai, China

[2]MoE Key Lab of Design and Electromagnetic Compatibility of High Speed Electronic System, and MoE Key Lab of Artificial Intelligence, AI Institute, Shanghai Jiao Tong University, Shanghai, China

Corresponding author: z.peng@sjtu.edu.cn

Supplementary Note 1: Phasor representation of multi-channel baseband signals

We elaborate on the phasor representation of multi-channel baseband signals with a simplified form. For ease of illustration, we first consider the single target scenario. With beat frequency demodulation, the phasor representation of the baseband beat signal of the i-th chirp with a certain receiver channel can be formulated as $A\exp\left[j(\varphi_R+\varphi_i)\right]$. Note that the signal amplitude $A(t)$ is generally not constant along fast time (i.e., during one chirp period) due to the hardware imperfection over a certain transmitting bandwidth. However, since it is essentially an ultra-low frequency amplitude modulation (i.e., making the ideal beat signal with a slowly changing amplitude envelope) and remains unchanged across multiple sweeps (i.e., slow time), we can ignore this factor. Therefore, for multiple receiver channels with a uniformly spaced linear antenna array, the phasor representation of multi-channel baseband signals of the i-th chirp can be formulated as

$$\begin{bmatrix} \mathrm{A}_1\exp\left(j\left(\varphi_R+\varphi_i\right)\right) \\ \mathrm{A}_2\exp\left(j(2\pi d\sin\theta/\lambda_c+\varphi_R+\varphi_i+\Delta\varphi_2)\right) \\ \vdots \\ \mathrm{A}_M\exp\left(j(2\pi(\mathrm{M}-1)d\sin\theta/\lambda_c+\varphi_R+\varphi_i+\Delta\varphi_M)\right) \end{bmatrix} \approx A\exp\left(j\varphi_R\right)\begin{bmatrix} \exp\left(j\varphi_i\right) \\ \exp\left(j(2\pi d\sin\theta/\lambda_c+\varphi_i)\right) \\ \vdots \\ \exp\left(j(2\pi(\mathrm{M}-1)d\sin\theta/\lambda_c+\varphi_i)\right) \end{bmatrix} \tag{1}$$

For real mmWave transceivers with multiple receiver antennas or multiple-input multiple-output (MIMO) virtual array, it inevitably encounters the problem of antenna gain and phase mismatches due to the imperfections in antenna layouts. In order to achieve accurate angle (including azimuth and elevation) estimation of the target, it is generally necessary to calibrate the mismatch because the mismatch leads to an ultra-low frequency modulation in angle-dimension and will produce a small deviation of incident angle estimation. However, it is worth noting that, for micro-motion sensing with the mmWBat, the amplitude and phase imbalances between multiple channels have a negligible influence on the phase evolution tracking due to the same effect on all chirp periods (i.e., the caused absolute phase shift is the same across multiple sweeps, which does not produce relative phase deviation for any two consecutive sweeps). Therefore, in the equation (1), we can assume $A_1\approx A_2\approx\cdots A_M$ and ignore the phase mismatch for mmWave micro-motion sensing.

Accordingly, we can extend to the full-field multi-target scenario. Note that the phasor representation corresponding to each target is terminally achieved with two consecutive demodulations from range- and angle-dimension, respectively. We quantify the micro-motions of full-field targets from the range-angle joint dimension.

Supplementary Note 2: Principle of clutter interference elimination

Description of problem of clutter interference

For mmWave sensing based applications (especially the micro-motion monitoring), the signal-to-noise ratio (SNR) is critical for achieving good performance. The total SNR is determined with the common thermal noise and the clutter interference. For these two noise sources, the former mainly cause small random errors, while the latter will cause large systematic errors, suggesting the key issue is the clutter interference elimination.

In a real mmWave sensing system, the range resolution is limited by the transmitted bandwidth. The interference of adjacent clutter (i.e., clutter reflected by adjacent objects) and aliasing clutter (i.e., clutter reflected by objects in a same range bin with the target of interest) are extremely possible or inevitable in practical applications. For ease of illustration, we take the scenario with two targets as an example. Here the corresponding baseband signal with two components (ignoring the amplitude variation along fast time) can be formulated as

$$S_B\left(iT+t\right)=a_1\,\mathrm{e}^{j\left(2\pi f_{b1}t+\varphi_{R1}+\varphi_{1i}\right)}+a_2\,\mathrm{e}^{j\left(2\pi f_{b2}t+\varphi_{R2}+\varphi_{2i}\right)} \tag{2}$$

The range profile and the demodulated beat frequency are commonly achieved by performing fast Fourier transform (FFT) of the baseband signal, with the corresponding N-point discrete Fourier transform derived as

$$\begin{aligned}S\left(f,i\right)&=\sum_{n=0}^{N-1}S_B\left(n\right)e^{-j2\pi fn\mathrm{T_s}}\\&=a_1e^{j\left(\varphi_{R1}+\varphi_{1i}\right)}\frac{\sin\left(\pi\left(f-f_{b1}\right)/N\right)}{\sin\left(\pi\left(f-f_{b1}\right)\right)}e^{-j\pi\left(f-f_{b1}\right)\left(N-1\right)}\\&+a_2e^{j\left(\varphi_{R2}+\varphi_{2i}\right)}\frac{\sin\left(\pi\left(f-f_{b2}\right)/N\right)}{\sin\left(\pi\left(f-f_{b2}\right)\right)}e^{-j\pi\left(f-f_{b2}\right)\left(N-1\right)}\end{aligned} \tag{3}$$

where N is the length of sampled baseband signal, and $f=k/\left(NT_s\right),\ k=0,1,\cdots N-1$ (T_s is the sampling interval) is the frequency bins. Since equation (3) has a very similar result with *sinc* functions, two components nearby in the frequency domain (i.e., $f_{b1}\approx f_{b2}$) will mix with each other due to the leak of *sinc* functions. The closer the two components are, the more they leak into each other. Hence, for the adjacent clutter and aliasing clutter interferences, with the current technique it is difficult to distinguish the adjacent beat frequency and impossible to distinguish two components within a same range bin. Furthermore, the key aspect of phase evolution tracking by beat frequency demodulation is severely distorted by another component due to mutual coupling, which is a tricky problem to be solved.

Here we analyze the first component as an example and illustrate the coupling interference problem. Usually, we can locate the corresponding beat frequency $\hat{f}_1$ by using peak detection of the

FFT amplitude spectrum (i.e., $\hat{f}_1 \approx f_{b1}$) and the desired initial phase information for the *i*-th chirp can be extracted as

$$\phi_{1i} = \arg\left[S\left(\hat{f}_1, i\right)\right] = \arg\left[a_1 e^{j(\varphi_{R1}+\varphi_{1i})} A e^{j\varphi_A} + a_2 e^{j(\varphi_{R2}+\varphi_{2i})} B e^{j\varphi_B}\right] \tag{4}$$

where $A = \sin\left(\pi\left(\hat{f}_1 - f_{b1}\right)/N\right) \big/ \sin\left(\pi\left(\hat{f}_1 - f_{b1}\right)\right)$, $B = \sin\left(\pi\left(\hat{f}_1 - f_{b2}\right)/N\right) \big/ \sin\left(\pi\left(\hat{f}_1 - f_{b2}\right)\right)$, $\varphi_A = \pi\left(f_{b1} - \hat{f}_1\right)\left(N-1\right)$, $\varphi_B = \pi\left(f_{b2} - \hat{f}_1\right)\left(N-1\right)$, and A and B are real numbers less than *N*. Note that when f_{b1} and f_{b2} have a big difference (i.e., the two targets are far apart), parameter B is very small (see *sinc* function) and can be ignored. However, for adjacent clutter and aliasing clutter interference scenarios, since $\hat{f}_1$, f_{b1} and f_{b2} have similar values, A and B have comparable values. Therefore, the phase history $\phi_{1i}\left(i = 1, 2, \cdots\right)$ is determined by two coupling components, which causes obvious systematic measurement error. The systematic error is mainly affected by the relative magnitude of a_1 and a_2, and the difference of f_{b1} and f_{b2}, which is challenging to quantitatively evaluate and mitigate in practical scenarios.

Interference elimination principle

For the bioinspired mmWave full-field micro-motion sensing approach, $S\left(\hat{f}_1, i\right)$ in (4) is equivalent to the estimated phasor obtained by the beat frequency demodulation. Therefore, the phasor corresponding to angle-dimension can be represented as

$$\begin{aligned} & a_1 A e^{j(\varphi_{R1}+\varphi_A)} \begin{bmatrix} \exp\left(j\varphi_{1i}\right) \\ \exp\left(j(2\pi d \sin\theta_1/\lambda_c + \varphi_{1i})\right) \\ \vdots \\ \exp\left(j(2\pi(\mathrm{M}-1) d \sin\theta_1/\lambda_c + \varphi_{1i})\right) \end{bmatrix} \\ & + a_2 B e^{j(\varphi_{R2}+\varphi_B)} \begin{bmatrix} \exp\left(j\varphi_{2i}\right) \\ \exp\left(j(2\pi d \sin\theta_2/\lambda_c + \varphi_{2i})\right) \\ \vdots \\ \exp\left(j(2\pi(\mathrm{M}-1) d \sin\theta_2/\lambda_c + \varphi_{2i})\right) \end{bmatrix} \end{aligned} \tag{5}$$

where θ_1 and θ_2 are the incident angles of target 1 and target 2, respectively. Due to the difference of incident angles, it is observed that the coupling phasor components of range-dimension are separated and isolated with the further demodulation of angle-dimension, offering an effective and novel approach to greatly mitigate the clutter interference. As a result, we can accurately extract the phase evolution based on the demodulation in range-angle joint dimension.

In addition, note that the obtained phasor corresponding to the desired component in range-dimension has a certain error with the ground truth because of the inaccurate demodulation frequency

(i.e., $\hat{f}_1 \neq f_{b1}$). This is inevitable due to the limited frequency resolution (see (3) and (4)), resulting in the estimated $\hat{f}_1$ generally having a small difference with f_{b1}. Fortunately, the phasor error only causes a constant phase shift (i.e., φ_A) for phase evolution tracking across all sweeps. Similarly, the calculated phasor error in angle-dimension also causes a constant phase shift, which also has no effect on the phase variation estimation of consecutive sweeps.

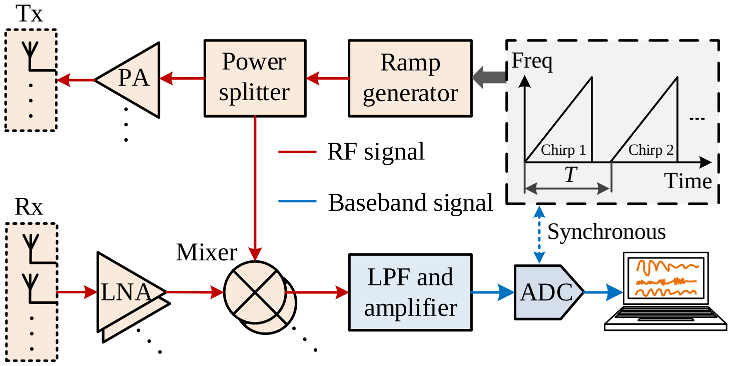

Supplementary Figure 1. Block diagram of the architecture of the mmWBat system.

Supplementary Fig. 1 shows a detailed system structure of the mmWBat system. The system consists of a mmWave transceiver with antenna array, an analogue-to-digital converter (ADC) and a laptop. The mmWave transceiver consists of a ramp generator, at least one power splitter, one power amplifier (PA), and multiple low-noise amplifiers (LNAs), mixers, baseband low-pass filters (LPFs) and amplifiers, and Tx/Rx (Tx: transmitter, Rx: receiver) antennas. The ramp generator produces linear-frequency-modulated continuous wave (LFMCW) radio-frequency (RF) signals, which is implemented with a phase-locked loop (PLL). The RF signal is divided into two paths with the power splitter component. The first portion is used for transmitting radio signals and the second portion as the local oscillator (LO) signal, which is used for mixing with the reflected signals from targets. According to the range correlation effect, the LO signal derived from the same source as the transmitted signal can effectively reduce the phase noise. The output of each mixer is low-pass filtered and amplified to produce the intermediate frequency (IF) baseband signal. Subsequently, the multi-channel baseband signals are digitized by the ADC and fed to the laptop for signal processing. To reserve the phase difference information, the multi-channel baseband signals need to be captured synchronously.

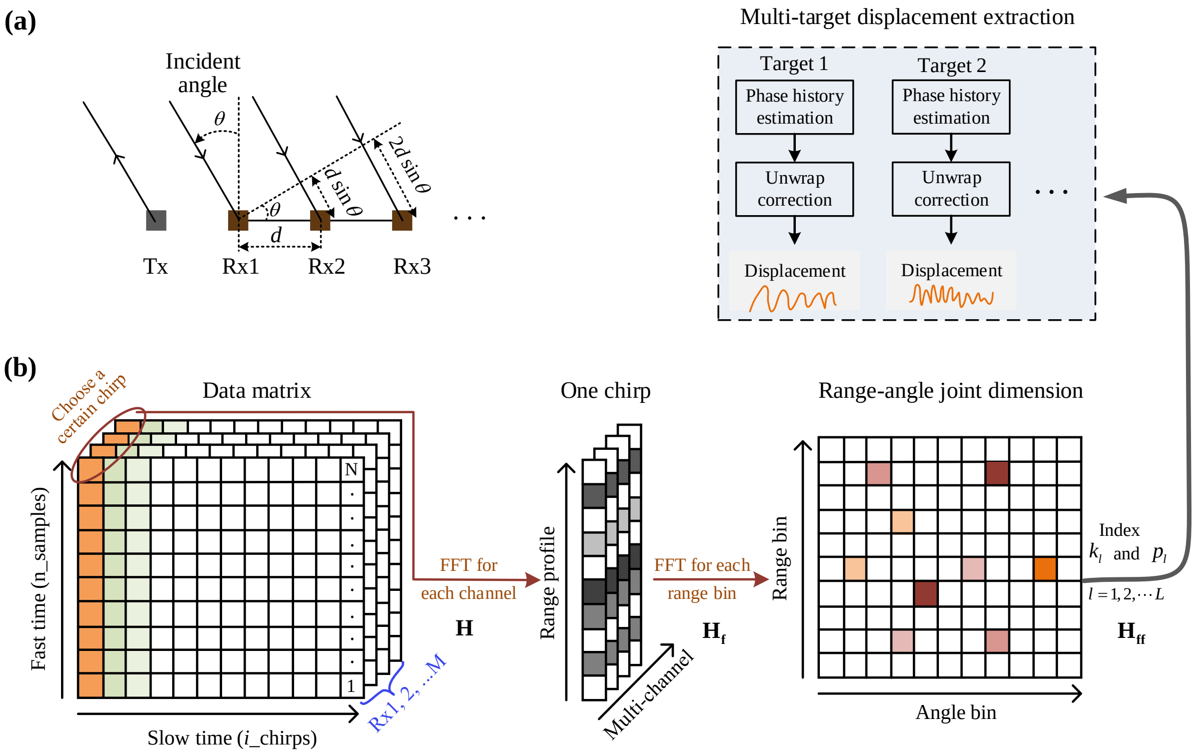

Supplementary Figure 2. Schematic of the basic algorithm of full-field phase evolution tracking. (a) Geometric relationship of the propagation paths for uniformly spaced multi-antenna linear array. **(b)** Procedures for implementing the algorithm of full-field phase evolution tracking. The different colors of the grid represent the different amplitudes or strengths of the components.

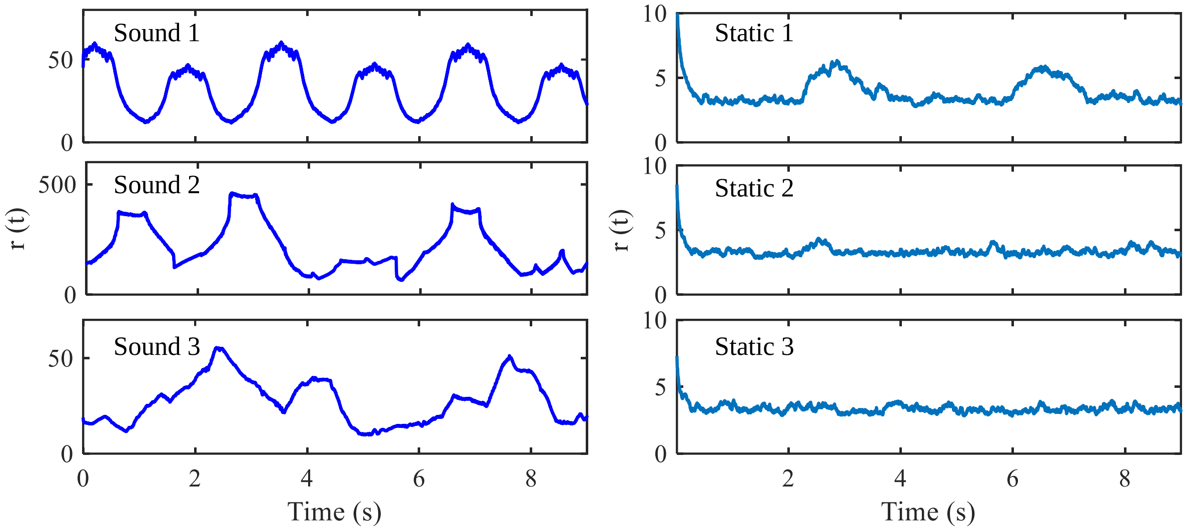

Supplementary Figure 3. Comparison results of the calculated ratio curves of the estimated phase evolution signals corresponding to three sound sources and three static objects in the RF microphone experiment. The sliding window length is 1 s, and the step is 2 ms. The obtained time-varying sparseness (TVS) indicators are 63, 427, 51 (left), 5.9, 4.2 and 4 (right), respectively.

Supplementary Video 1. Multi-person noncontact vital sign monitoring.

Supplementary Video 2. Full-field vibration measurement of two targets with three typical scenarios.

Supplementary Video 3. Example of full-field dynamic monitoring of a scaled bridge.

Supplementary Video 4. Multi-source sound recovery of RF microphone.
